# American Association of Obstetricians and Gynecologists

**Published:** 1906-10

**Authors:** 


### American Association of Obstetricians and Gynecologists.

THE nineteenth annual meeting of this association was held at Cincinnati, September 20-22, under the presidency of Dr. John Young Brown, of Saint Louis. The attendance of members and visitors was numerous, the debates were spirited, and the enthusiasm, beginning with the opening session on Thursday morning, never abated until the final adjournment at five o'clock Saturday afternoon. It was the universally expressed opinion that the association never appeared in better form, nor held a better meeting from whatever viewpoint the judgment could be taken.

It is a notable fact that in spite of sweltering atmospheric conditions the debates on this occasion were of an unusually spirited character, the meeting room being crowded to the doors on several occasions. The number of former presidents in attendance was twelve,—a remarkable showing,—the whole number being seventeen. Of these, three are dead,—Rohé, Davis, and Dunning,—one has resigned, one has held office two terms, leaving but one who did not attend. Dr. Vander Veer, of Albany, was detained at home by unavoidable causes but sent his paper with a letter of explanation expressing great regret that he could not be present.

To the president, Dr. John Young Brown, of Saint Louis, no small degree of credit is due for the large attendance, as well as for the admirable manner in which he conducted the meeting. In the latter function he was most ably assisted by the first vice-president, Dr. James N. West, of New York. The committee of arrangements, too, must not be forgotten in our estimate of the causes of success. Led by the chairman, Dr. Charles L. Bonifield, with the secretary, Dr. Magnus A. Tate, a close second, it would be difficult to conceive of a better organised or administered committee than this one at Cincinnati. Every item that could contribute to the completeness of administration or to the convenience of the members was provided, leaving no unprotected points, no neglected details.

In enumerating the several factors which contributed to the success of the meeting, the Hotel Havlin must not be overlooked. This elegant new establishment, under the management of Mr. James T. Clyde, proved admirably adapted to the purposes of such a meeting, and nothing was omitted by Mr. Clyde and his staff that could make for convenience or comfort.

The place of meeting next year was left to the executive council to name. The officers elected were: president, Robert Tuttle Morris, of New York; vice-presidents, George W. Crile, of Cleveland, and Charles Lybrand Bonifield, of Cincinnati; secretary, William Warren Potter, of Buffalo; treasurer, Xavier Oswald Werder, of Pittsburg; executive councillors, Hugo Otto Pantzer, vice L. H. Dunning, deceased,—Rufus Bartlett Hall, to succeed himself, and John Young Brown, vice Morris, elected president.

